# Duration of Untreated Psychosis, Treatment Response, and Resting State Functional Connectivity in Antipsychotic-Naïve First-Episode Psychosis Patients

**DOI:** 10.1093/schizbullopen/sgag007

**Published:** 2026-04-24

**Authors:** Hillary N Patton, Jose O Maximo, Adrienne C Lahti

**Affiliations:** Department of Psychology, University of Alabama at Birmingham, Birmingham, AL, USA; Department of Psychiatry and Behavioral Neurobiology, University of Alabama at Birmingham, Birmingham, AL, USA; Department of Psychiatry and Behavioral Neurobiology, University of Alabama at Birmingham, Birmingham, AL, USA

**Keywords:** brain networks, early intervention, mediation analysis, psychotic disorders

## Abstract

**Background:**

Meta-analyses consistently link longer duration of untreated psychosis (DUP) to worse clinical outcomes, but the underlying neurobiological mechanisms remain unclear. This study examined whether DUP influences resting-state functional connectivity (FC) and if FC mediates the relationship between DUP and treatment response following antipsychotic drug (APD) treatment.

**Hypotheses:**

We hypothesized that longer DUP would be associated with both reduced FC and worse treatment response, and that FC would mediate the relationship between DUP and response to antipsychotic treatment.

**Study Design:**

One hundred antipsychotic-naïve first- episode psychosis patients underwent resting state fMRI. We extracted signal from regions within the default mode (posterior cingulate), salience (anterior cingulate), and central executive (right posterior parietal cortex) networks, correlating them with whole-brain activity. Associations between DUP and FC were examined while controlling for age, sex, and framewise displacement. Mediation analyses tested whether FC of these networks mediated the relationship between DUP and treatment response.

**Study Results:**

Findings indicated that longer DUP was associated with reduced functional connectivity in all networks of interest. Further, we found that functional connectivity of the central executive and salience networks mediated the relationship between DUP and treatment response.

**Conclusions:**

These results suggest that there are pathophysiological processes inherent in the DUP. These results are also consistent with the possibility that brain network connectivity is a neurological link between longer DUP and poor treatment response. Our data emphasize the importance of early intervention targeting DUP’s adverse effects on the brain.

## Introduction

Longstanding evidence suggests that much of the clinical and psychosocial deterioration in psychosis spectrum disorders occurs within the first few years of the illness,^1–3^ highlighting the importance of early intervention and limiting the duration of untreated psychosis (DUP) during the first episode of psychosis (FEP). DUP refers to the time between psychosis onset and the initiation of appropriate treatment^4,5^ and is considered a critical factor in treatment outcomes.^6^ In the United States, the median DUP is approximately 74 weeks, or about 17 months.^7^ Meta-analyses have consistently found that longer DUP is linked to poorer clinical outcomes,^8–11^ including worse symptomatic and functional recovery,^12^ greater cognitive impairment,^13^ reduced response to treatment,^8^ more severe positive and negative symptoms,^14^ and a higher risk of relapse.^15^

Neurobiological research implicates widespread disturbances in functional brain connectivity in psychosis^16^ with dysconnectivity in large-scale networks such as the default mode (DMN), central executive (CEN), and salience (SN) thought to underlie symptoms.^17–19^ Although findings vary, a general trend emerges of reduced connectivity within and between these networks in psychosis.^20,21^ This dysconnectivity may begin during the prodromal phase and worsen throughout the illness course, further supporting the value of early intervention.^22^

Altered FC in these brain networks may underlie the relationship between DUP and illness trajectory.^23^ However, findings are mixed.^23–26^ For example, our previous study with an overlapping sample of patients found that longer DUP is associated with reduced functional connectivity in the default, central executive, and salience networks.^23^ Sarpal, Robinson^24^ also found a negative correlation between DUP and functional connectivity between various cortical regions and the striatum. In contrast, others have found no relationship between DUP and resting state connectivity.^25,26^ Discrepant findings may reflect methodological differences, including prior exposure to antipsychotics, variability in illness severity at assessment, and small sample sizes.^4,27^

Despite mixed evidence, reducing DUP remains a key target for early intervention, given its modifiable nature and strong association with improved outcomes.^11^ Shorter DUP is linked to greater neuronal plasticity and better response to intensive remediation efforts,^28^ lower risk of a multi-episode course of illness,^29^ and decreased risk of relapse.^8,30,31^ Longer DUP is also associated with poor response to medications and worse trajectories of positive and negative symptoms over time.^32^ When looking specifically at antipsychotic treatment response, a meta-analysis highlighted that shorter DUP is associated with greater antipsychotic response, as measured by improvement in positive symptom severity, global psychopathology, and negative symptom severity.^8^

To date, there are only a handful of studies that have established a link between brain connectivity, DUP, and antipsychotic treatment response.^23,24,33^ In our previous study, we found that FC in the DMN mediated the relationship between DUP and treatment response.^23^ Building on our previous work and using a larger, overlapping sample of antipsychotic-naïve first episode psychosis patients, the goal of this imaging study was two-fold: (1) to examine the relationship between DUP and FC in the DMN, CEN, and SN and (2) to test whether FC mediates the relationship between DUP and antipsychotic treatment response. Based on our earlier findings, we hypothesized that longer DUP would be associated with reduced response to treatment and altered FC. Next, we hypothesized that FC would mediate the relationship between DUP and treatment response.

## Methods and Materials

### Participants

First-episode antipsychotic naïve psychosis patients were recruited from the emergency department, outpatient clinics, and inpatient services within the Department of Psychiatry at the University of Alabama at Birmingham. Exclusion criteria were MRI contraindications, diagnosable central nervous system illnesses, pregnancy, major medical conditions (e.g., traumatic brain injury, seizure disorders, stroke, cephalic disorders, brain tumors, etc.), and active substance dependence (except nicotine). Patients with a history (within the past year) of alcohol or drug abuse (except nicotine and cannabis) were also excluded. Prior to study entry patients were either antipsychotic-naïve or had no more than five days of lifetime antipsychotic exposure. Approval for this study was granted by the University of Alabama at Birmingham’s ethics committee, and written informed consent was obtained for all participants. There were 119 medication-naïve patients with FEP and 19 were excluded after quality control (e.g., invalid scan or excessive motion), leaving us with a total sample size of 100.

Over the course of 16 weeks, patients underwent treatment with risperidone with a flexible dosing schedule. Starting at 1 to 2 mg, risperidone was titrated in 1 to 2-mg increments, with dosing based on therapeutic effect and side effects. One patient was prescribed olanzapine. Concomitant medications were permitted based on clinical need, and they were prescribed following the MRI session. During the trial, the following concomitant medications were prescribed for 62 medication-naïve FEP patients: sertraline (*n* = 33), trazadone (*n* = 28), lithium (*n* = 1), benztropine (*n* = 3), lorazepam (*n* = 13), valproic acid (*n* = 1), amphetamine (*n* = 1), diazepam (*n* = 1), fluoxetine (*n* = 7), bupropion (*n* = 8), escitalopram (*n* = 6), citalopram (*n* = 1), lisdexamfetamine (*n* = 1), buspirone (*n* = 1), divalproex sodium (*n* = 1), antipsychotic drug injectable (*n* = 1), and paliperidone (*n* = 1). 32 patients were taking more than one psychiatric medication. Medication compliance was assessed via pill counting at each visit and the time between scanning and antipsychotic initiation was <24 hours.

### Clinical Assessment

Diagnoses were made in consensus following DSM-5 criteria by two board-certified psychiatrists from all available direct assessment and historical information. DUP was determined by asking patients and their families to provide a general timeline for symptom onset and type(s) of symptoms they were experiencing. To ensure accuracy, patients and caregivers were asked at multiple time points when their psychosis symptoms first began, and this information was corroborated with any available medical records. If responses were discrepant, clinical judgement was utilized to determine the most credible and accurate date of onset. For the current study, DUP was operationally defined as the time between initial onset of active phase positive symptoms and the initiation of antipsychotic treatment (e.g., when the first antipsychotic prescription was written).

The Brief Psychiatric Rating Scale (BPRS) was used to assess symptom severity.^34^ In concurrence with the literature,^35–37^ treatment response was calculated as the percent change in BPRS positive symptoms from the initial study visit to week 16, since patients with first-episode psychosis may require up to 16 weeks to show a response. The minimum BPRS scores were also accounted for in the formula. It was calculated as [(BPRS positive baseline –BPRS positive week 16)/(BPRS positive baseline – BPRS positive minimum)] x 100.

### Data Acquisition

Imaging for the proposed study was acquired via a 3T whole-body Siemens MAGNETOM Prisma MRI Scanner equipped with a 20-channel head coil. A high-resolution T1-weighted structural scan was acquired for anatomical reference (MPRAGE: TR = 2400 ms; TE = 2.22 ms; inversion time = 1000 ms; flip angle = 8°; GRAPPA factor = 2; voxel size = 0.8 mm3). Resting state functional MRI data were acquired in opposing phase encoding directions (anterior > posterior and posterior > anterior; TR = 1550 ms; TE = 37.80 ms; flip angle = 71°, FOV = 104 mm^2^; multi-band acceleration factor = 4; voxel size= 2 mm^3^; 225 volumes). Participants underwent ~12-minute resting fMRI scan in which they were asked to keep their eyes open and stare passively at a fixation cross and let their mind wander.

### Data Preprocessing

The first 10 volumes on each fMRI scan were discarded to allow for signal equilibration. Susceptibility artifacts were corrected using spin echo field maps in FSL’s top-up, and then the two corrected fMRI runs were combined resulting in a single 4-dimentional image of 430 total volumes.^38^ Data preprocessing was conducted in CONN functional connectivity toolbox version 20.b.^39^ Preprocessing consisted of slice-timing and motion correction, structural coregistration, normalization to the Montreal Neurological Institute (MNI) space, low-bandpass filtering (0.008 < *f* < 0.08 Hz), and spatial smoothing with a 4-mm full width at half maximum Gaussian kernel, and artifact detection and removal with ART-based scrubbing (compositive volume-to-volume motion > 0.5 mm and intensity >3 Standard Deviations). Framewise displacement and percentage of censored data were calculated. Motion outliers were identified using the Artifact Detection Tools toolbox (NeuroImaging Tools and Resources Collaboratory) and then censored (composite volume-to-volume motion >0.5 mm and intensity >3 SDs). Additionally, six motion parameters derived from rigid-body realignment and their derivatives, as well as the first component time series derived from CSF and white matter masks using aCompCor within the CONN toolbox and corresponding derivatives were regressed out from the signal.

### Statistical Analyses

Study variables were inspected for missingness and the percentage of cases with missing data was 26% while the percentage of missing data points was approximately 8.6%, with all missing data coming from the dependent variable, treatment response. Little’s Missing Completely at Random (MCAR) test yielded non-significance, indicating that the mechanism of missingness is Missing Completely at Random (MCAR); χ^2^(10) = 6.243, *p* = .794. An expectation maximization algorithm was utilized in order to ensure complete data for participants on all variables of interest.

The preprocessed data were analyzed in CONN functional connectivity toolbox version 20.b. The analysis examined the correlation between functional connectivity among three brain networks and DUP (log transformed to address positively skewed data distribution). Three CONN toolbox-based regions of interest were used: default mode network (seed region: posterior cingulate cortex), salience network (seed region: anterior cingulate cortex), and central executive network (seed region: right posterior parietal cortex). Seed regions were selected based on prior literature identifying these areas as core hubs of their respective networks^40–43^ and in accordance with the our laboratory’s previous studies.^23,44^ DUP was correlated with functional connectivity while statistically controlling for age, sex, and framewise displacement as covariates of no interest. Individual whole-brain z-transformed correlation maps were created for each region of interest to each voxel in the brain. Complementary analyses constrained to a priori network masks were also conducted; however, no clusters survived standard cluster-level correction, so whole-brain corrected results are reported. All analyses were corrected using voxel (*p_uncorrected_* <.01) and cluster-level and false discovery rate corrections (*p_FDR_* <.05).

Hayes’ PROCESS macro for SPSS was used to conduct a mediation analysis, which allowed for the estimation of the indirect effect of functional connectivity of any significant cluster within the three brain networks of interest (DMN, CEN, and SN) on the relationship between DUP and treatment response. Any significant cluster values obtained in CONN were extracted for the mediation analysis and each significant cluster was tested for possible mediation.

## Results

### Sample and Clinical Characteristics

Sample characteristics are summarized in Table 1. Participants ranged from 15-40 in age (*M* = 23.14, *SD* = 5.59) and had an average of approximately 13 years of education. The sample was 60% male and predominantly non-Hispanic Black (57%), aligning with regional census data for Birmingham, Alabama. The most common diagnoses included schizophrenia (46%), schizoaffective disorder (20%), and psychosis not otherwise specified (17%). Most participants were nonsmokers (67%) and over half reported cannabis use (55%).

**Table 1:** Demographic and Clinical Variables.

Variable		M	SD
Age		23.14	5.59
Education		12.97	2.34
Parental SES^a^		5.46	4.94
Smoking, Packs per Day		0.21	0.36
Duration of Untreated Psychosis		20.43	34.70
Treatment Response		79.30	26.99
BPRS baseline			
	Total	47.88	11.55
	Positive	14.73	4.06
	Negative	5.49	3.07
BPRS Week 16			
	Total	28.88	6.23
	Positive	6.01	2.81
	Negative	5.21	2.48
Risperidone dose at week 16, mg		4.78	2.28
Scan Quality Data			
	Volumes after scrubbing, %	91.72	7.65
	Framewise displacement, mm	0.18	0.08
		**n**	**%**
Sex	Female	40	40.0
	Male	60	60.0
Race	Non-Hispanic Black	57	57.0
	Non-Hispanic white	37	37.0
	Hispanic/Latinx	1	1.0
	Asian	3	3.0
	Other	2	2.0
Diagnosis	Schizophrenia	46	46.0
	Bipolar disorder with psychosis	4	4.0
	Schizophreniform disorder	7	7.0
	Psychosis NOS	17	17.0
	Brief psychotic disorder	4	4.0
	MDD with psychosis	2	2.0
	Schizoaffective disorder	20	20.0
Smoking Status	Smoker	32	32.0
	Non-smoker	67	67.0
Cannabis Use	Cannabis User	55	55.0
	Non-Cannabis User	45	45.0

Abbreviations: BPRS, Brief Psychiatric Rating Scale; MDD with psychosis, major depressive disorder with psychosis; mg, milligram; mm, millimeter; NOS, not otherwise specified

^a^Ranks determined from Diagnostic interview for Genetic Studies (1-18 scale); Lower numerical value (higher rank) corresponds to higher socioeconomic status.

Descriptive characteristics for the primary study variables, clinical measures and data quality information are presented in Table 1. On average, the duration of untreated psychosis was 20.43 months. Participants showed a substantial treatment response (*M* = 79.30%) with total BPRS scores decreasing from 47.88 at baseline to 28.88 at week 16. Positive symptoms scores showed the largest reduction over time, while negative symptoms scores remained relatively stable. The average risperidone dose at week 16 was 4.78 mg.

### Partial Correlation Analyses

For the partial correlation analyses, significant clusters and their voxel coordinates and size can be found in Table 2. All partial correlation results are presented in Table 3. For the DMN, we identified one significant cluster showing a negative correlation between DUP and baseline posterior cingulate cortex (PCC) FC in the right middle frontal gyrus (*r*(95) = -0.49, *p* < .001, two-sided). For the CEN, we found three significant clusters showing a negative correlation between DUP and baseline right posterior parietal cortex FC. These included left occipital cortex (*r*(95) = -0.55, *p* < .001, two-sided), left middle frontal gyrus (*r*(95) = -0.47, *p* < .001, two-sided), and right lateral occipital cortex (*r*(95) = -0.46, *p* < .001, two-sided). For the SN, we found five significant clusters showing a negative correlation between DUP and baseline anterior cingulate (ACC) cortex FC. These clusters included the right lateral occipital cortex (*r*(95) = -0.47, *p* < .001, two-sided), left middle temporal gyrus (*r*(95) = -0.44, *p* < .001, two-sided), left lateral occipital cortex (*r*(95) = -0.44, *p* < .001, two-sided), right precentral gyrus (*r*(95) = -0.37, *p* < .001, two-sided), and right supramarginal gyrus (*r*(95) = -0.37, *p* < .001, two-sided).

**Table 2:** Brain Regions Showing Significant Correlations Between DUP and Network FC.

		MINI Coordinates				
Seed (Network)	Location	x	y	z	Cluster Size	*t* statistic
PCC (DMN)	Middle Frontal Gyrus, R	+46	+32	+38	204	−5.48
ACC (SN)	Lateral Occipital Cortex, Inferior Division, R	+50	−60	−12	360	−5.23
	Middle Temporal Gyrus, Temporooccipital, L	−52	−60	−10	206	−4.86
	Lateral Occipital Cortex, Inferior Division, L	−48	−74	+04	211	−4.80
	Precentral Gyrus, R	+58	+04	+36	144	−3.91
	Supramarginal Gyrus, Anterior Division, R	+56	−28	+56	102	−3.95
RPPC (CEN)	Lateral Occipital Cortex, Superior Division, L	+08	−74	+40	340	−6.56
	Middle Frontal Gyrus, L	−38	+22	+56	187	−5.23
	Lateral Occipital Cortex, Inferior Division, R	+44	−72	−14	120	−5.09

Abbreviations: CEN, central executive network; DMN, default mode network; DUP, duration of untreated psychosis; FC, functional connectivity; L, left; LPPC, left posterior parietal cortex; MNI, Montreal Neurological Institute; PCC, posterior cingulate cortex; R, right; RPPC, right posterior parietal cortex; SN, salience network

**Table 3:** Partial Correlations between DUP and Functional Connectivity.

Variable	*n*	*M*	*SD*	1	2	3	4	5	6	7	8	9	10
1. DUP	100	.76	.74	-									
2. DMN	100	.07	.10	−.49^***^	-								
3. CEN 1	100	.07	.11	−.55^***^	.48^***^	-							
4. CEN 2	100	.15	.12	−.47^***^	.45^***^	.35^***^	-						
5. CEN 3	100	−0.06	.08	−.46^***^	.33^***^	.50^***^	.35^*^	-					
6. SN 1	100	.03	.09	−.47^***^	.35^***^	.33^***^	.25^*^	.32^***^	-				
7. SN 2	100	.04	.10	−.44^***^	.27^**^	.25^*^	.33^***^	.22^*^	.74^***^	-			
8. SN 3	100	.04	.10	−.44^***^	.28^**^	.31^**^	.22^*^	.30^**^	.80^***^	.63^***^	-		
9. SN 4	100	.14	.14	−.37^***^	.34^***^	.22^*^	.27^**^	.21^*^	.71^***^	.67^***^	.57^***^	-	
10.SN 5	100	.14	.13	−.37^***^	.29^**^	.22^*^	.24^*^	.09	.74^***^	.72^***^	.59^***^	.67^***^	-

Partial correlations between duration of untreated psychosis (DUP) and significant mean z-score cluster values of functional connectivity within the default mode (DMN), central executive (CEN), and salience networks (SN) while controlling for age, sex, and framewise displacement. ^*^  *p* < .05, ^**^  *p* < .01, ^***^  *p* < .001.

### Mediation Analyses

**Figure 1:**
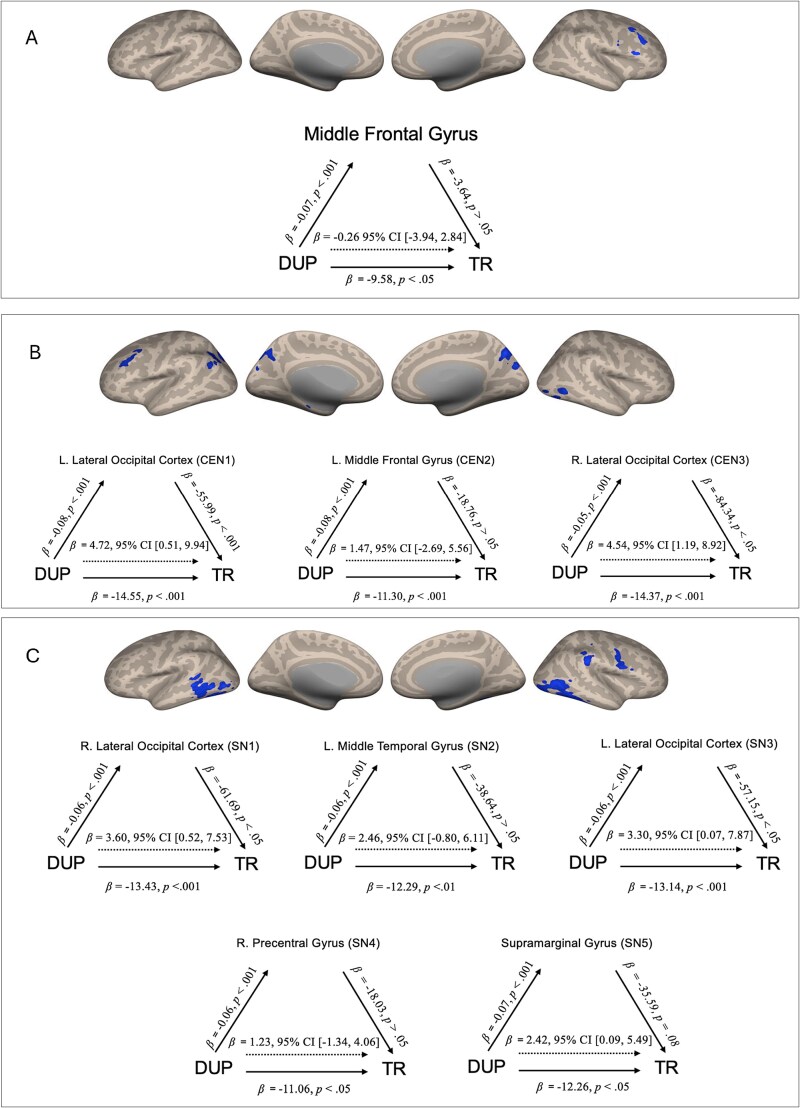
Mediation models for the default mode (A), central executive (B), and salience networks (C). Models of duration of untreated psychosis as a predictor of treatment response, mediated by functional connectivity of default mode network, salience network, and central executive network clusters. The confidence interval for the indirect effect is a BCa bootstrapped CI based on 10000 samples. The dashed lines represent the indirect effects while the solid lines represent the direct effects. CEN, central executive network; DMN, default mode network; DUP, duration of untreated psychosis; SN, salience network; TR, treatment response.

Results of the significant mediation analyses are presented in Figure 1. Across all models, longer DUP was significantly associated with reduced functional connectivity at baseline (path a), with βs ranging from -0.05 to -0.08 (*p* < .001). DUP also significantly predicted poorer treatment response (path c’), accounting for approximately 7% of the variance, with *β*s ranging from -9.58 to -14.55.

Default mode network connectivity did not mediate the DUP-treatment response relationship (See Figure 1A). While DUP was significantly associated with reduced DMN functional connectivity (*β* = -0.07, *p* < .001), neither the association between DMN connectivity and treatment response (*β* = -3.64, *p* = .89) nor the indirect effect (*β* = -0.26, 95% BCa CI [-3.94, 2.84]) was significant. Within the central executive network, two of the three clusters (CEN 1 and CEN 3) significantly mediated the relationship between DUP and treatment response (See Figure 1B). Indirect effects were small but significant **(***β* = 4.72, 95% BCa CI [0.51, 9.94]; *β* = 4.54, 95% BCa CI [1.19, 8.92]; both *Bs* = 0.13). Each of these clusters also showed significant direct associations between reduced functional connectivity and lower treatment response (*β* = -55.99 and -84.34, *p* < .05). In the salience network (See Figure 1C), three of five clusters (SN 1, SN 3, and SN 5) significantly mediated the effect of DUP on treatment response, with small but significant indirect effects (*β*s = 3.60, 3.30, and 2.42; 95% BCa CIs [0.52, 7.53], [0.07, 7.87], and [0.09, 5.49]; *B*s = 0.10, 0.09, and 0.07). These clusters also demonstrated significant associations between reduced FC and poorer treatment response (*β*s = -61.69 and -57.15 *p* < .05).

To address potential confounding effects of cannabis use, an additional analysis was conducted (see supplemental material) excluding participants who endorsed cannabis use (n = 45). The pattern of results remained highly consistent with the primary analysis, with continued associations between longer DUP and reduced functional connectivity and small but significant mediation effects within the central executive and salience networks. Default mode network connectivity did not mediate the relationship between DUP and treatment response.

#### Mediation Analyses Summary

Across all analyses, the direct effect of DUP on functional connectivity (path a) was significant, with increasing DUP associated with reduced functional connectivity. Additionally, the direct effect of DUP on treatment response (path c’) was significant, with approximately 7% of the variance in treatment response (see supplementary material) accounted for by DUP. Increasing DUP was associated with reduced response to treatment. Functional connectivity within three out of five salience network clusters and two out of three central executive network clusters significantly mediated the relationship between DUP and treatment response. All significant mediation effects were small.

## Discussion

The present study examined the relationship between DUP and functional brain network connectivity within a large sample of medication-naïve first-episode psychosis patients. The present study also assessed the mediating role of functional connectivity of three key brain networks on the relationship between DUP and antipsychotic treatment response. Results demonstrated that longer DUP was associated with reduced functional connectivity in all partial correlation analyses after controlling for age, sex, and framewise displacement.

Overall, these findings are consistent with only a handful of studies examining the relationship between resting state functional connectivity and DUP. For example, Manivannan, Foran^45^ found that DUP was correlated with decreased functional connectivity within the dorsolateral prefrontal cortex. In our previous work utilizing a smaller overlapping sample, we similarly found that DUP was negatively correlated with functional connectivity within the DMN, SN, and CEN.^23^ Additionally, the work of Sarpal, Robinson^24^ reported that greater DUP was associated with lower functional connectivity in striatal, frontal, and parietal regions.

Taken together, our results highlight a general pattern of lower functional connectivity (within increasing DUP) in brain regions associated with higher cognitive functions such as language and memory, processing sensory and visual information, and enabling successful motor control. Specifically, the middle temporal gyrus, MFG, and supramarginal gyrus are involved with language comprehension, semantic memory processing, as well as literacy.^46–48^ The lateral occipital cortex is essential to visual processing and has specialized functions related to the integration of visual stimuli and perception formation.^49,50^ Further, both the supramarginal gyrus and precentral gyrus relate to motor function and sensory integration.^51^ The inverse relationship between functional connectivity and DUP in the current study results suggest a breakdown in these cognitive processes secondary to worsening psychosis.

Our data also demonstrated that functional connectivity mediates the relationship between DUP and antipsychotic treatment response, suggesting brain network connectivity as a neurological basis of the relationship between longer DUP and unfavorable clinical outcomes. Specifically, we were able to demonstrate that functional connectivity of the salience network and central executive network mediates the relationship between DUP and antipsychotic treatment response. These results are consistent with the possibility that brain network connectivity is a neurological link between longer DUP and poor treatment response.

There are a limited number of studies examining the mediating role of functional connectivity on the relationship between DUP and TR. We previously found that DMN functional connectivity^23^ mediated the relationship between DUP and TR. Additionally, Sarpal, Robinson^24^ found that corticostriatal connectivity acted as a mediator between DUP and TR. Shorter DUP is linked with better outcomes, which suggests that antipsychotic medications may mitigate the pathophysiological mechanisms inherent in the DUP. Taken together, these findings suggest that brain functional and anatomical connectivity play an important role in treatment response and may be a neurobiological foundation between DUP and treatment response.

Our study includes several strengths and limitations. First, we included a large sample of medication-naïve first episode psychosis patients. Since most patients had no prior exposure to antipsychotic medications before the scan, we were able to minimize potential confounding effects related to medication use. Additionally, although this study shares a portion of its sample with our prior work, it includes 45 additional participants and examines the effects of treatment. Importantly, by using Hayes PROCESS macro with bootstrapping for the mediation analysis, we were able to more precisely estimate indirect effects, providing further insight into the relationships among DUP, resting state functional connectivity, and treatment response.

Next, duration of untreated psychosis is a retrospective estimate of how long the patient has been experiencing psychosis symptoms. As such, recall bias is a possibility, especially as patients who are acutely or severely psychotic may have more difficulty with long-term recall.^52^ However, we obtained DUP using a clinical interview and operationally defined the onset of treatment as the initiation of antipsychotic medication prescription. A review by Register-Brown and Hong^53^ assessing methods of determining DUP noted that clinical interviews are just as reliable as standardized assessment tools and that defining treatment onset as first hospitalization or first-ever antipsychotic prescription may have better validity when compared to other methods. Further, to ensure accuracy of the DUP estimate, patients and caregivers were asked at multiple timepoints throughout the study. Additionally, we chose not to exclude patients on the basis of cannabis use. Although cannabis use may impact brain structure and function,^54^ it is considered a substantial risk factor for the development of a psychotic disorder^55^ and thus has great clinical relevance. Excluding patients with a history of cannabis use would have biased our sample and impacted the generalizability of our findings. Finally, it is worth noting that the present study focused on the default mode network (DMN), salience network (SN), and central executive network (CEN) based on the triple network model,^43^ which proposes that disruptions within and between these three core networks are foundational to a wide range of psychiatric disorders,^56–58^ including psychosis. These networks support higher-order cognitive processes^23^ and dysfunction in one network can propagate to the others.^43^ Prior work demonstrates widespread dysconnectivity among the DMN, SN, and CEN in psychosis,^20,59^ with abnormalities emerging during the prodromal period and worsening across first-episode and chronic phases.^60^ Given this evidence, and the relevance of DUP to early illness progression, our analyses targeted these theoretically and empirically established networks. We acknowledge, however, that other resting-state networks may also be implicated in DUP, and future research should extend beyond the triple network model to examine broader network-level effects.

## Conclusion

In sum, the current study used resting state functional magnetic resonance imaging to test hypotheses about alterations that are associated with DUP. The results have the potential to aid in early detection and diagnosis efforts as aberrant connectivity patterns within these networks may serve as biomarkers for identifying individuals at risk for developing psychosis. The current study also has the potential to inform treatment targets. For example, pharmacological and non-pharmacological interventions aimed at modulating disruptions in network connectivity could be developed to address symptoms or prevent further illness progression.

The current study sought to address methodological constraints of small sample size, variation of illness, and level of antipsychotic treatment within previous research by using a large sample of medication-naïve first-episode psychosis patients. Overall, the current study aimed to contribute to a better understanding of the neurobiological mechanisms underlying psychosis, which has the potential to inform early intervention strategies to improve outcomes and quality of life for individuals with psychosis-spectrum disorders.

## Supplementary Material

sgag007_Supplementary_materials

## Data Availability

The datasets generated during and/or analyzed during the current study are available in the NIMH Data Archive, https://nda.nih.gov/edit_collection.html?id=2916, NCT 034420101.

## Clinical Trial Registration

Trajectories of Treatment Response as Window into the Heterogeneity of Psychosis: A Longitudinal Multimodal Imaging Study, NCT03442101 https://clinicaltrials.gov/ct2/show/NCT03442101

Glutamate, Brain Connectivity and Duration of Untreated Psychosis (DUP), NCT02034253 https://clinicaltrials.gov/ct2/show/NCT02034253
