# Supplementary material for: Duration of Untreated Psychosis, Treatment Response, and Resting State Functional Connectivity in Antipsychotic-Naïve First-Episode Psychosis Patients
Source: Schizophr Bull Open. 2026 Apr 24;7(1):sgag007. doi: 10.1093/schizbullopen/sgag007 (PMC13131233; doi:10.1093/schizbullopen/sgag007)
Supplement: sgag007_Supplementary_materials [file sgag007_supplementary_materials.docx]

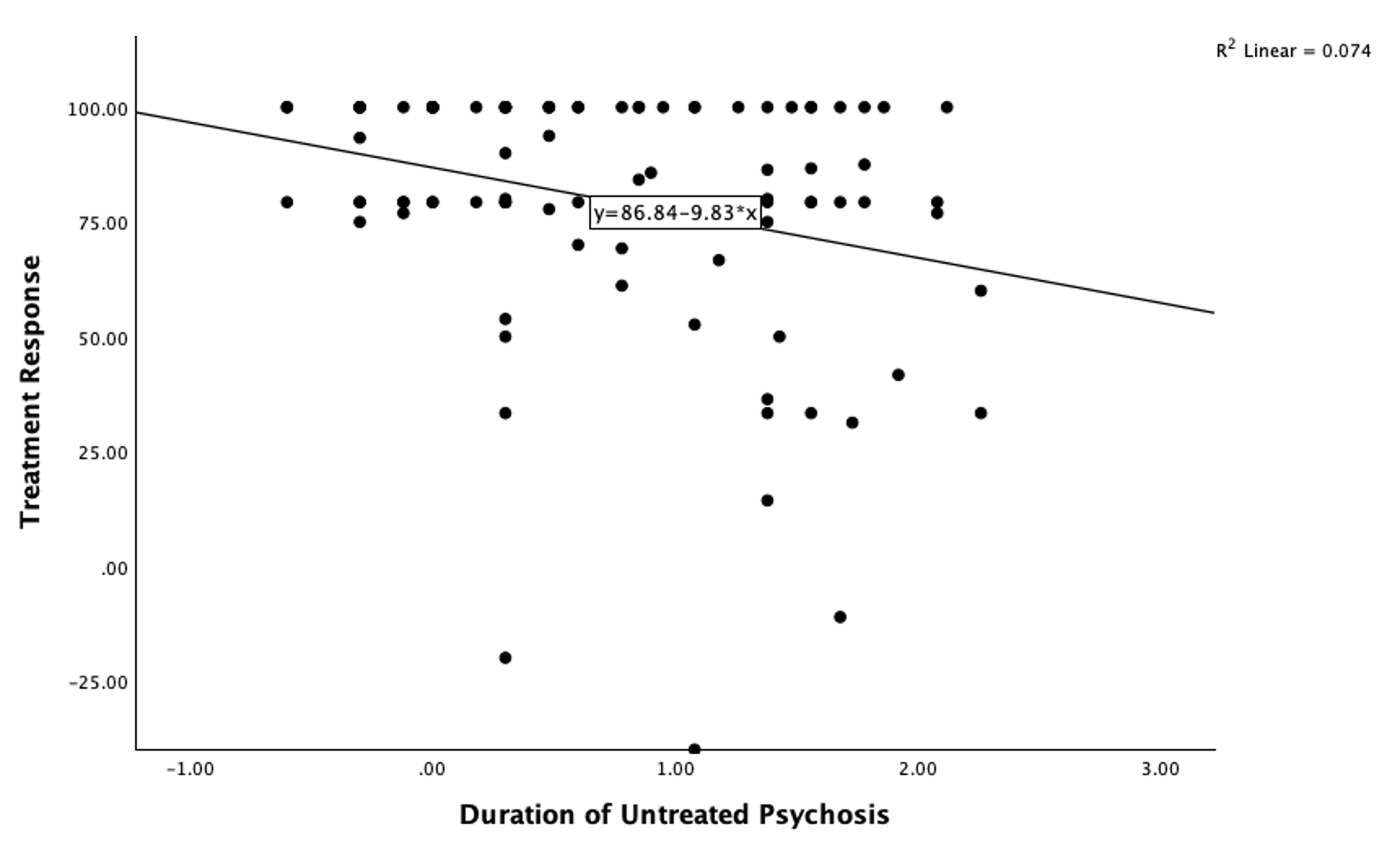


Supplemental Figure: Scatterplot illustrating the association between duration of untreated psychosis (DUP; log10 transformed) and treatment response. The fitted linear regression line is shown. DUP accounted for approximately 7% of the variance in treatment response (R^2^ = .07).

____________________________________________________________________________

To address potential confounding effects of active cannabis use, a supplemental analysis was conducted excluding participants who endorsed cannabis use. This resulted in a reduced sample (n = 45). All analyses were conducted using the same procedures as described in the methods of the manuscript.

Results (see table below) were highly consistent with the primary findings. Within the DMN, we identified one significant cluster demonstrating a negative correlation between DUP and baseline posterior cingulate cortex (PCC) FC in the right middle frontal gyrus, replicating the location observed in the full sample. Within the CEN, four significant clusters showed negative associations between DUP and baseline right posterior parietal cortex. These clusters were located in the left lateral occipital cortex, right intracalcarine cortex, and two clusters within the right lateral occipital cortex. Within the SN, three significant clusters emerged, located in the right lateral occipital cortex, left lateral occipital cortex, and precentral gyrus.

Consistent with the original analyses, default mode network connectivity did not significantly mediate the relationship between DUP and treatment response. With regards to the CEN, mediation effects continued to remain significant with two of the clusters. Both clusters demonstrated significant indirect effects (*β* = 6.76, 95% BCa CI [0.64, 15.24]; *β* = 6.52, 95% BCa CI [1.20, 14.69]; both *Bs* = 0.18) as well as significant direct associations between reduced functional connectivity and lower treatment response (*β* = -99.47 and -135.69, *p* < .05). Similarly, within the salience network, two clusters significantly mediated the effect of DUP on treatment response (*β* = 7.83, 95% BCa CI [1.67, 16.56]; *β* = 8.18, 95% BCa CI [1.43, 18.03]; both *Bs* = 0.22). One of the SN clusters demonstrated a significant association between reduced FC and poorer treatment response (*β* = -101.79, *p* < .05) while the other cluster trended towards significance (*β* = -91.92, p = 0.054).

In sum, exclusion of participants with active cannabis use did not substantively alter the pattern of findings. The association between longer DUP and reduced functional connectivity remained evident, and significant mediation effects persisted within both the central executive and salience networks. As in the full sample, mediation effects were small in magnitude but statistically significant.

**Supplemental Table.** **Brain Regions Showing Significant Correlations Between DUP and Network FC in a reduced sample (n = 45)**

|  |  | MINI Coordinates | | |  |  |
| --- | --- | --- | --- | --- | --- | --- |
| Seed (Network) | Location | x | y | z | Cluster Size | *t* statistic |
| PCC (DMN) | Middle Frontal Gyrus, R | +56 | +28 | +24 | 229 | -5.24*** |
| ACC (SN) | Lateral Occipital Cortex, Inferior Division, R | +50 | -60 | -12 | 1147 | -4.05*** |
|  | Lateral Occipital Cortex, Inferior Division, L | -48 | -72 | +02 | 983 | -5.55*** |
|  | Precentral Gyrus, R | +58 | +04 | +34 | 163 | -5.15*** |
| RPPC (CEN) | Lateral Occipital Cortex, Superior Division, L | -24 | -74 | +36 | 1465 | -6.58*** |
|  | Lateral Occipital Cortex, Inferior Division, R | +32 | -50 | -14 | 963 | -5.56*** |
|  | Intracalcarine Cortex, R | +18 | -64 | +04 | 238 | -3.80*** |
|  | Lateral Occipital Cortex, Superior Division, R | +28 | -70 | +24 | 170 | -3.97*** |
| CEN, central executive network; DMN, default mode network; DUP, duration of untreated psychosis; FC, functional connectivity; L, left; MNI, Montreal Neurological Institute; PCC, posterior cingulate cortex; R, right; RPPC, right posterior parietal cortex; SN, salience network; *, *p* < .001 | | | | | |  |
